# A Simple HPLC/DAD Method Validation for the Quantification of Malondialdehyde in Rodent’s Brain

**DOI:** 10.3390/molecules26165066

**Published:** 2021-08-21

**Authors:** George Jîtcă, Erzsébet Fogarasi, Bianca-Eugenia Ősz, Camil Eugen Vari, Amelia Tero-Vescan, Amalia Miklos, Mădălina-Georgiana Bătrînu, Carmen Maria Rusz, Mircea Dumitru Croitoru, Maria Titica Dogaru

**Affiliations:** 1 Department of Pharmacology and Clinical Pharmacy, Faculty of Pharmacy, George Emil Palade University of Medicine, Pharmacy, Science and Technology of Târgu Mureș, 540139 Târgu Mureș, Romania; george.jitca@umfst.ro (G.J.); bianca.osz@umfst.ro (B.-E.Ő.); camil.vari@umfst.ro (C.E.V.); maria_dogaru2004@yahoo.com (M.T.D.); 2 Doctoral School of Medicine and Pharmacy, I.O.S.U.D., George Emil Palade University of Medicine, Pharmacy, Science and Technology of Târgu Mureș, 540139 Târgu Mureș, Romania; batrinumadalina@yahoo.com; 3 Department of Toxicology and Biopharmacy, Faculty of Pharmacy, George Emil Palade University of Medicine, Pharmacy, Science and Technology of Târgu Mureș, 540139 Târgu Mureș, Romania; mircea.croitoru@umfst.ro; 4 Department of Biochemistry, Faculty of Pharmacy, George Emil Palade University of Medicine, Pharmacy, Science and Technology of Târgu Mureș, 540139 Târgu Mureș, Romania; amelia.tero-vescan@umfst.ro (A.T.-V.); amalia.miklos@umfst.ro (A.M.); 5 Faculty of Pharmacy, George Emil Palade University of Medicine, Pharmacy, Science and Technology of Târgu Mureș, 540139 Târgu Mureș, Romania; carmenrusz20@gmail.com

**Keywords:** oxidative stress, malondialdehyde, brain, liquid chromatography, diode array detector

## Abstract

In the present study, a HPLC/DAD method was set up to allow for the determination and quantification of malondialdehyde (MDA) in the brain of rodents (rats). Chromatographic separation was achieved on Supelcosil LC-18 (3 μm) SUPELCO Column 3.3 cm × 4.6 mm and Supelco Column Saver 0.5 μm filter by using a mobile phase acetonitrile (A) and phosphate buffer (20 mM, pH = 6) (B). Isocratic elution was 14% for (A) and 86% for (B). The injection volume (loop mode) was 100 μL with an analysis time of 1.5 min. Flow rate was set at 1 mL/min. The eluted compound was detected at 532 nm by a DAD detector by keeping the column oven at room temperature. The results indicated that the method has good linearity in the range of 0.2–20 μg/g. Both intra- and inter-day precision, expressed as RSD, were ≤15% and the accuracies ranged between ±15%. The lower limit of quantification (LLOQ), stability, and robustness were evaluated and satisfied the validation criteria. The method was successfully applied in a study of chronic toxicology following different treatment regimens with haloperidol and metformin.

## 1. Introduction

Oxidative stress is currently one of the most intensely studied processes. It appears when reactive oxygen species (ROS) and reactive nitrogen species (RNS) production exceeds the neutralizing capacity of endogenous antioxidant systems [1,2]. Mitochondrial impairment constitutes a crucial and critical factor in the aging process and development of age-related disorders and is generally accepted. This impairment occurs due to the loss of oxidative phosphorylation capacity and oxygen radical leakage, with the subsequent apparition of ROS. These reactive species can constitute either physiological signals (low levels) or toxic species (high levels) that affect cellular integrity, depending on the concentration [3,4]. One of the most studied reactions involved in the oxidative stress is lipid peroxidation, thus the increased reactivity of these ROS and RNS makes all biological structures susceptible to oxidative processes, particularly the brain [5], due to its high lipid content [6]. Lipid peroxidation reactions affect the cellular membrane, degrading it, and subsequently, degradation products are obtained, i.e., malondialdehyde (MDA) [7,8], which can propagate and amplify the oxidative lesions. Compared to other degradation compounds, MDA is obtained in high quantities and is considered a marker of oxidative stress [9,10]. MDA’s property of cross-linking other molecules contributes to its toxic potential, at the same time, the mutagenic and carcinogenic effects are attributed to the chemical bonds that MDA forms with the nitrogen bases from the nucleic acid structure [11].

**Figure 1:**
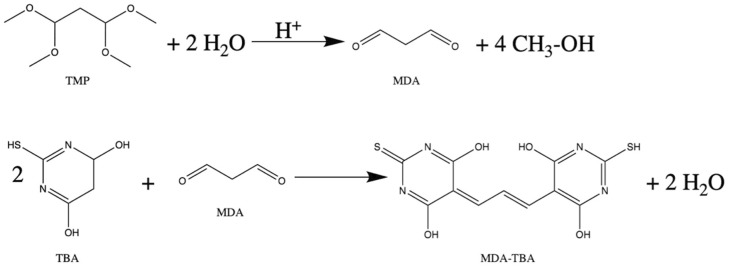
Reaction of generative malondialdehyde (MDA) from 1,1,3,3-Tetramethoxypropane (TMP) and the colored pink product resulted from the reaction between thiobarbituric acid (TBA) and MDA.

Increased levels of MDA in the brain have been observed in central nervous system (CNS) disorders [3,12,13], such as Alzheimer’s disease [14], Parkinson’s disease [15], or in cases of consumption or abuse of drugs [16]. A widely known and used method of MDA assay is the spectrophotometric method in which a pink colored complex is obtained (MDA-TBA) from the MDA reaction with thiobarbituric acid (TBA) under high temperature and low pH, as shown in Figure 1.

Despite attempts of optimization of this method, spectrophotometric determination still has limitations due to TBA that can interact with other compounds such as carbohydrates, amino acids, and certain pigments, resulting in higher values [17,18] that are undesirable, especially in human tissue determinations in which more specific methods are required [19]. Thus, in an attempt to avoid the bias of the TBA interaction with other molecules and with the purpose of obtaining MDA values as close as possible to the real values, the present paper aims to validate a method of identifying MDA in the brain. Separation of the analytes was obtained using a high-performance liquid chromatographic (HPLC) system coupled with a diode array detector (HPLC/DAD). This technique overcomes the limitations of the spectrophotometric method in terms of specificity and sensitivity, being a simple, fast, and cost-effective method.

## 2. Results and Discussion

### 2.1. Method Validation

#### 2.1.1. Optimization of Sample Preparation

In order to be as accurate as possible, three methods of sample preparation were tested. In the first method, we used an automatic homogenizer IKA Ultra-Turrax Tube Drive. For the second method, manual trituration of the sample in mortar with pestle was used in the presence of silicon dioxide. The third method combined the above-mentioned methods. Comparing the areas of the peaks obtained by the three processing methods, the following values were obtained: the mean of the three obtained areas under the curve (AUC), 65538.33; standard deviation (SD), 7584.11; and relative standard deviation (RSD%), 11.57. No major differences were observed between the three methods used, in terms of areas. In order to avoid unnecessary prolongation of processing, the automated method was used with the IKA Ultra-Turrax Tube Drive.

#### 2.1.2. Chromatographic Conditions

For MDA analysis, chromatographic separation was performed using a mobile phase acetonitrile (A) and phosphate buffer (20 mM, pH = 6) (B). Isocratic elution was 14% for (A), 86% for (B). The injection volume (loop mode) was 100 μL with an analysis time of 1.5 min. Flow rate was set at 1 mL/min, eluent was monitored with a DAD, and the best chromatogram achieved was at 532 nm, using Supelcosil LC-18 (3 μm) SUPELCO Column 3.3 cm × 4.6 mm and Supelco Column Saver 0.5 μm filter [20].

#### 2.1.3. Linearity and LLOQ

**Figure 2:**
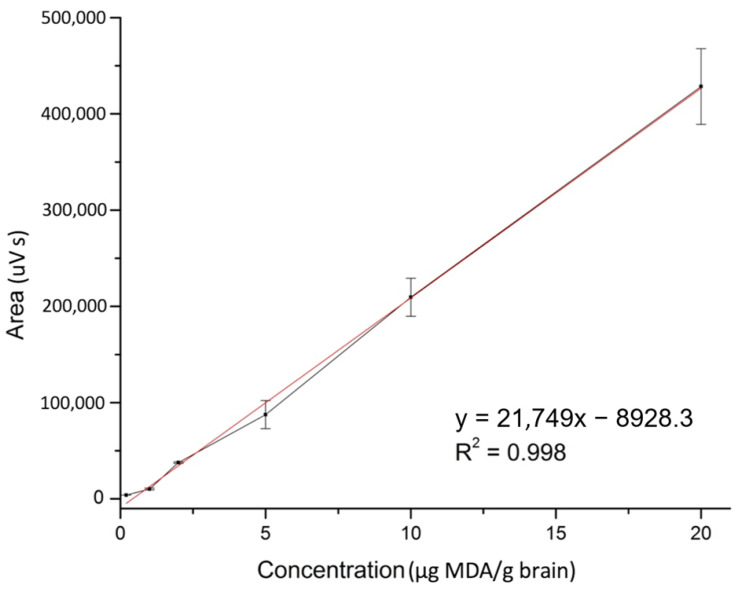
Analytical curve of the HPLC/DAD assay of malondialdehyde (MDA) in the range of 0.20–20 µg/g brain, with line equation y = 21,749x − 8928.3.

The linearity of the method was verified through the analytical curve using six levels of concentrations, evaluated in triplicates. The analytical curve was described by the linear equation: y = 21,749x − 8928.3 while the regression coefficient was r^2^ = 0.998, as illustrated in Figure 2, where y is the analyte peak area ratio and x is the concentration (μg/g) as shown in Table 1.

#### 2.1.4. Selectivity

The selectivity of the method in terms of its ability to accurately measure the analyte of interest in the presence of other components that were present in the sample matrix was demonstrated by the analysis of blank matrices. To verify the selectivity, we injected three blank samples prepared according to the sample preparation procedure described in Section 3.3. *Sample preparation* with the following modification: TBA without MDA, MDA without TBA, and brain sample without TBA in which the reagent was replaced with purified water. After injecting these blank samples in neither one of these cases, interferences did not occur at the retention time of 1.1 min. The peak purity in all cases was above 98.7%.

#### 2.1.5. Accuracy

Quality control (QC) samples at lower limit of quantification (LLOQ) concentration and three different concentration levels (low, medium, and high) were spiked for the determination of precision and accuracy. Five replicates for each level of QC samples were assayed in one run for the intra-day procedure.

**Figure 3:**
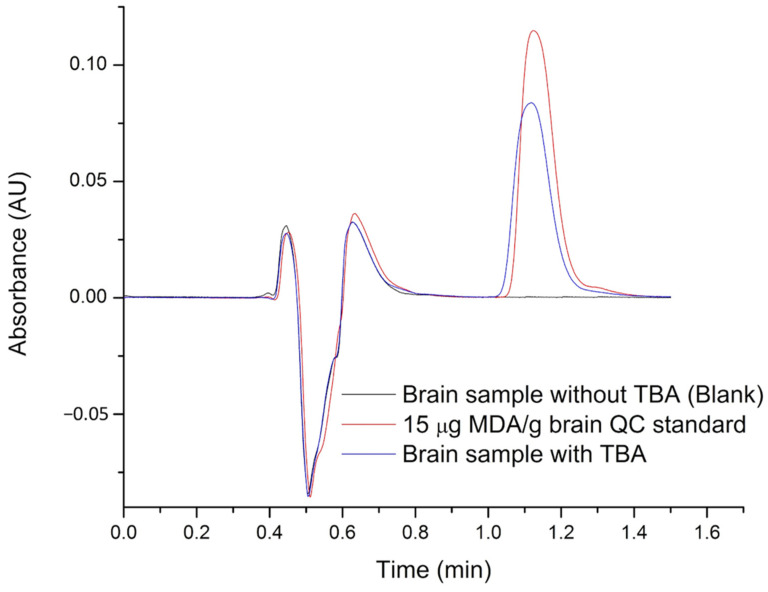
Representative chromatogram of a rat brain sample spiked. Analysis conditions: mobile phase (1 mL/min), acetonitrile: phosphate buffer (14:86), C18 column, detection: DAD at 532 nm, room temperature.

The accuracy was evaluated based on the percentage of MDA recovered from the brain matrix. Representative chromatogram of MDA is illustrated in Figure 3. Data for the intra- and inter-day accuracy for MDA at LLOQ and three QC levels are illustrated in Table 2.

#### 2.1.6. Precision

Inter-day precision was evaluated on two different days using five replicates for each level of QC samples and at LLOQ concentration. Results of precision (intra-day and inter-day) were expressed as RSD%. Data for the intra- and inter-day precision for MDA at LLOQ and three QC levels are illustrated in Table 2.

Both within run and between runs precision (RSD%) of the QC samples were ≤15%, and the accuracy ranged between ±15%. These results demonstrated that the method is reproducible for the determination of MDA in a rodent’s brain as the results demonstrated that the precision and accuracy are in acceptable limits.

#### 2.1.7. Stability

The stability was analyzed by assaying the frozen (−80 °C) QC samples with the QC samples kept at 25 °C for 24 h and for 48 h, both in triplicates. Analytical recovery varied between 106.69–115.79% after 24 h and between 100.45–114.05% after 48 h at −80 °C. For the samples that have been kept under room temperature, the recovery varied between 98.09–109.06% after 24 h and between 93.26–110.96% after 48 h. The data are listed in Table 3.

#### 2.1.8. Robustness

**Figure 4:**
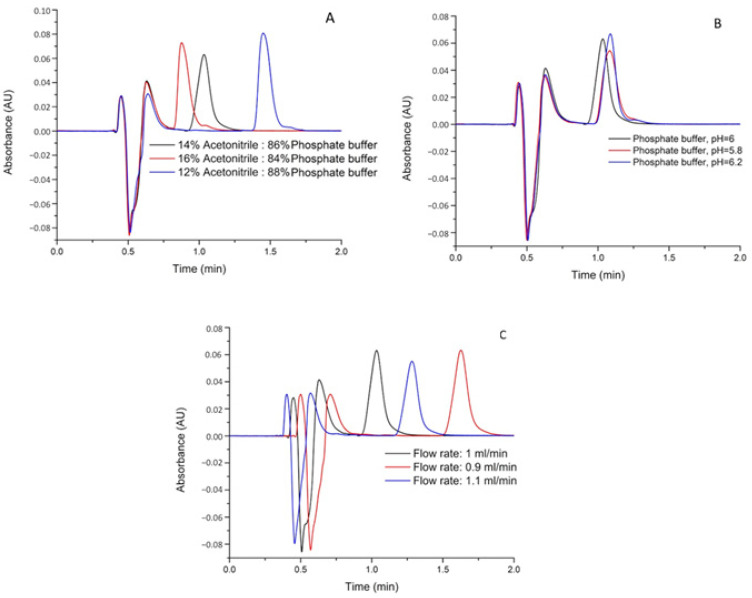
Changes of malondialdehyde (MDA) retention time depending on the variation of the chromatographic parameter (**A**) represents the variation of the mobile phase composition; (**B**) represents the variation of the mobile phase pH; and (**C**) represents the variation of the mobile phase flow rate).

The robustness of the method was assessed with the performance of variations in three crucial chromatographic parameters (mobile phase ratio, pH value of mobile phase, and flow rate). All assays were performed at a concentration level of 0.5, 7.5, and 15 µg/g for MDA, in five replicates. All the data are listed in Table 4. Changes in retention times as a function of variation of the chromatographic parameter are illustrated in Figure 4.

## 3. Materials and Methods

### 3.1. Chemical and Reagents

All chemicals and reagents were analytically pure, and they were purchased from different providers: acetonitrile (VWR International, S.A.S., Fonteney-sous-Bois, France), anhydrous disodium phosphate (Na₂HPO₄), and 85% phosphoric acid solution (H_3_PO_4_) were purchased from Merck KGaA (Darmstadt, Germany). Thiobarbituric acid (98%) and phosphate buffer solution (PBS) were purchased from Sigma-Aldrich (Darmstadt, Germany). Sulfuric acid solution (H_2_SO_4_) (96%) was purchased from Chemical Company (Iasi, Romania). For the preparation of MDA standard solutions, 1,1,3,3-Tetramethoxypropane (TMP, 99%, Sigma Aldrich, Shanghai, China) was used [21,22]. Ultra pure water was obtained using a Milli-Q purification system (Merck Millipore Corporation, Burlington, MA, USA).

### 3.2. Preparation of Solutions

For MDA stock solution was prepared by diluting 460 μL of TMP in 100 mL ultra pure water; the concentration of this solution was equivalent to the MDA solution of 2 mg/mL. Standard work solutions at 9 concentration levels were prepared by diluting stock solutions with ultra pure water. Six triplicate samples for linearity (0.2–20 ug/g brain) and three QC samples were prepared at 0.5, 7.5, and 15 ug/g brain. For each QC sample, the analysis was performed in five replicates.

### 3.3. Sample Preparation

Twenty male rats weighing 450–500 g were individually housed in plastic cages, maintained on a 12:12 h light-dark cycle, and fed ad libitum. All animals were decapitated under anesthesia with ketamine and xylazine in a dose mixture of ketamine (100 mg/kg) and xylazine (10 mg/kg), in order to collect the brain samples. The brains were rapidly removed, immediately frozen in liquid nitrogen, and stored at −80 °C until analysis. For MDA analysis, brains were homogenized in IKA Ultra-Turrax Tube Drive and were subsequently divided in equal quantities. Afterward, 1 g of brain sample was spiked with 10 μL of working solution, and then PBS was added in a three times higher volume than the sample volume. Samples were vigorously vortexed for 1 min, and immediately after, samples were centrifuged (10,000× *g* for 10 min). After centrifugation, acetonitrile (ACN) was added for protein precipitation (1:3, *v*/*v*). The samples were centrifuged (10,000× *g* for 10 min) and the collected supernatant was diluted with pure water (1:1, *v*/*v*). A volume of 600 μL TBA (4 mg/mL) and 1000 μL sulfuric acid (2.66 μL/mL) were added to 400 μL sample, followed by heating at 100 °C for 60 min in TS-100C, Thermo-Shaker (BioSan, Riga, Latvia). After heating, the samples were transferred in HPLC vials and analyzed shortly after the derivatization reaction.

### 3.4. Instrumentation

Liquid chromatography analysis was performed on a Merck HPLC system: quarternary pump Merck Hitachi L-7100, auto sampler Merck Hitachi L-7200, column thermostat Merck Hitachi L-7360, DAD Merck Hitachi L-7455, interface Merck Hitachi L-7000, solvent degaser Merck Hitachi L-7612, software D-7000 HSM-Manager using Supelcosil LC-18 (3 μm) SUPELCO Column 3.3 cm × 4.6 mm, and Supelco Column Saver 0.5 μm filter.

### 3.5. Method Validation

In the present study, the validation method was performed in accordance with the regulatory guidelines (FDA 2018). Chosen validation parameters were linearity, selectivity, accuracy, precision, lower limit of quantification (LLOQ), stability, and robustness.

### 3.6. Study Application

In addition, in order to demonstrate the applicability of analytical methods, a study of chronic CNS toxicity was performed on 40 rodents (rats), which were randomly divided into 4 groups comprising 10 rats each (Control, Haloperidol, Metformin, and Haloperidol + Metformin). The treatment consisted of distilled water, haloperidol 2 mg/kg metformin 500 mg/kg, and haloperidol 2 mg/kg + metformin 500 mg/kg in a volume of 1 mL/kg for 40 days, administered through an oral feeding cannula. At the end of the study, all the rodents were decapitated under anesthesia with ketamine and xylazine in a dose mixture of ketamine (100 mg/kg) and xylazine (10 mg/kg), in order to collect the brain samples. The brains were removed, frozen in liquid nitrogen, stored at −80 °C, and afterward, were analyzed with the method presented in this study.

### 3.7. Ethical Considerations

All procedures were conducted in compliance with all experimental procedures in accordance with European Directive 2010/63/EU and was approved by the Ethics Committee for Scientific Research of the George Emil Palade University of Medicine, Pharmacy, Science and Technology of Târgu Mureș (approval no. 533/2019) and by the National Sanitary Veterinary and Food Safety Authority (approval no. 42/2020).

## 4. Conclusions

Analytical curves for MDA in the brain were linear for the concentration range of 0.2–20 μg/g, with a regression coefficient of r^2^ = 0.998. This validation method demonstrates good accuracy and precision in accordance with regulatory guidelines [23]. According to these, the accuracy must comprise the ±15% interval and the precision must be ≤15%. Regarding LLOQ, accuracy must comprise the interval of ±20% and the precision must be ≤20%.

The method is suitable for MDA quantification from a rodent’s brain and in studies that aim for the measurement and estimation of oxidative stress in different treatments and induced pathologies. Unlike the classic spectrophotometric methods [24,25], this method is superior in terms of sensitivity and specificity; the interferences of other compounds capable of absorption at 532 nm in VIS is avoided. Moreover, this method is simple and cost-effective; it does not imply the multiple steps of preparation for analytical extraction that may lead to interactions. Instead, a derivatization reaction is proposed.

## Figures and Tables

**Table 1:** Analytical factors of the HPLC method.

Analytical Factor	MDA
LLOQ (µg/g brain)	0.20
rLLOQ (%)	94.91
LLOQrec (µg/g brain)	0.20
rLLOQrec (%)	104.97
Slope	21749
Y-intercept	8928.30
Determination coefficient (r^2^)	0.998
Analytical range (µg/g brain)	0.20–20
Retention time (min)	1.11 ± 0.01

rLLOQ, relative lower limit of quantification; LLOQrec, recovery-corrected LLOQ; rLLOQrec (%), relative recovery-corrected LLOQ.

**Table 2:** Precision and accuracy of malondialdehyde (MDA) in quality control samples.

Conc. (µg/g Brain)		Intra-Day				Inter-Day			
		Mean	SD	RSD %	Accuracy %	Mean	SD	RSD %	Accuracy %
MDA	0.2	0.18	0.03	15.30	90.42	0.17	0.02	14.30	83.88
	0.5	0.49	0.06	13.02	98.18	0.50	0.06	11.17	99.08
	7.5	6.90	0.52	7.52	91.99	7.00	0.43	6.20	93.40
	15	15.36	0.67	4.39	102.38	15.10	0.65	4.27	100.67

**Table 3:** Stability assessment for samples stored at −80 °C and at room temperature for 24 and 48 h, respectively.

Parameters		Stability for Stored Samples at −80 °C						Stability for Samples Stored at Room Temperature					
		Conc. (µg/g Brain)						Conc. (µg/g Brain)					
		0.5		7.5		15		0.5		7.5		15	
		24 h	48 h	24 h	48 h	24 h	48 h	24 h	48 h	24 h	48 h	24 h	48 h
MDA	Mean	0.52	0.55	7.98	7.55	16.76	15.42	0.53	0.54	7.15	6.43	15.06	14.67
	Rec *, %	106.69	114.05	115.79	109.49	109.15	100.45	109.06	110.96	103.71	93.26	98.09	95.58
	SD	0.08	0.06	0.06	0.13	0.52	0.54	0.05	0.07	0.74	0.31	0.67	0.90
	RSD%	3.56	2.63	0.60	1.44	2.78	3.16	1.99	2.76	8.19	3.70	3.97	5.43

* Recovery, average of three concentrations.

**Table 4:** Robustness of the method by variation of three chromatographic parameters (mobile phase ratio, pH value of mobile phase, and flow rate).

	Conc. (µg/g Brain)	Mean Area (mV S) ± RSD, %	Retention Time (min) ± RSD, %	Peak Purity (%) ± RSD, %
Mobile phase ratio (*v*/*v*), acetonitrile: buffer (pH 6)				
84:16	0.5	11.18 ± 0.58	0.88 ± 1.32	97 ± 1.57
	7.5	149.41 ± 1.13	0.87 ± 0.66	98 ± 1.56
	15	304.56 ± 1.44	0.87 ± 0.01	99 ± 0.59
86:14	0.5	9.13 ± 12.87	1.12 ± 0.52	98 ± 1.02
	7.5	152.88 ± 7.15	1.11 ± 0.90	98 ± 1.56
	15	332.64 ± 3.56	1.11 ± 0.52	97 ± 1.19
88:12	0.5	10.54 ± 0.29	1.44 ± 0.40	98 + 1.02
	7.5	164.09 ± 0.97	1.43 ± 0.01	98 ± 1.56
	15	317.87 ± 1.91	1.43 ± 0.01	97 ± 1.19
Mobile phase pH value				
5.8	0.5	9.42 ± 0.33	1.09 ± 0.92	98 ± 1.02
	7.5	157.13 ± 1.42	1.08 ± 0.01	96 ± 1.59
	15	308.76 ± 3.16	1.08 ± 0.66	97 ± 1.58
6	0.5	9.13 ± 12.87	1.12 ± 0.52	98 ± 1.02
	7.5	152.88 ± 7.15	1.11 ± 0.90	98 ± 1.56
	15	332.64 ± 3.56	1.11 ± 0.52	97 ± 1.19
6.2	0.5	9.63 ± 1.11	1.09 ± 0.01	98 ± 1.17
	7.5	148.03 ± 3.49	1.10 ± 1.33	97 ± 1.03
	15	303.40 ± 0.16	1.09 ± 0.99	97 ± 1.57
Flow rate (mL/min)				
0.9	0.5	9.24 ± 3.30	1.63 ± 0.35	98 ± 0.59
	7.5	173.82 ± 2.29	1.63 ± 0.35	97 ± 1.57
	15	349.94 ± 1.04	1.64 ± 0.35	98 ± 1.02
1.0	0.5	9.13 ± 12.87	1.12 ± 0.52	98 ± 1.02
	7.5	152.88 ± 7.15	1.11 ± 0.90	98 ± 1.56
	15	332.64 ± 3.56	1.11 ± 0.52	97 ± 1.19
1.1	0.5	9.80 ± 2.49	1.29 ± 0.92	98 ± 0.59
	7.5	148.52 ± 1.22	1.28 ± 0.01	98 ± 1.02
	15	293.04 ± 1.34	1.30 ± 0.92	98 ± 0.59

## Data Availability

The datasets that support the findings of this study are available from the corresponding authors upon reasonable request.

## Sample Availability

Samples of the compounds are not available from the authors.
